# Supplementary material for: Machine Learning Accelerates Crystallization for Structure Determination
Source: Angew Chem Int Ed Engl. 2026 May 4;65(25):e1218503. doi: 10.1002/anie.1218503 (PMC13266958; doi:10.1002/anie.1218503)

checkCIF/PLATON report

Structure factors have been supplied for datablock(s) lcz080

THIS REPORT IS FOR GUIDANCE ONLY. IF USED AS PART OF A REVIEW PROCEDURE FOR PUBLICATION, IT SHOULD NOT REPLACE THE EXPERTISE OF AN EXPERIENCED CRYSTALLOGRAPHIC REFEREE.

No syntax errors found. CIF dictionary Interpreting this report

Datablock: lcz080

Bond precision:	C-C = 0.0075 A		Wavelength=1.54184
Cell:	a=8.6364(2)	b=13.0948(2)	c=14.9126(4)
	alpha=103.145(2)	beta=96.009(2)	gamma=98.251(2)
Temperature:	100 K		

	Calculated	Reported
Volume	1608.72(7)	1608.72(6)
Space group	P -1	P -1
Hall group	-P 1	-P 1
Moiety formula	C25 H10 Ag3 F18 N7 O2	C15 H3 Ag3 F18 N6, C10 H7 N O2
Sum formula	C25 H10 Ag3 F18 N7 O2	C25 H10 Ag3 F18 N7 O2
Mr	1106.01	1106.01
Dx,g cm-3	2.283	2.283
Z	2	2
Mu (mm-1)	15.868	15.868
F000	1056.0	1056.0
F000’	1061.17	
h,k,lmax	10,16,18	10,16,18
Nref	6864	6513
Tmin,Tmax	0.186,0.205	0.079,1.000
Tmin’	0.033	

Correction method= # Reported T Limits: Tmin=0.079 Tmax=1.000
AbsCorr = MULTI-SCAN

Data completeness= 0.949   Theta(max)= 77.880

R(reflections)= 0.0389(6148)   wR2(reflections)= 0.1104(6513)

S = 1.054   Npar= 524

The following ALERTS were generated. Each ALERT has the format
test-name_ALERT_alert-type_alert-level.
Click on the hyperlinks for more details of the test.

Alert level C

```
PLAT042_ALERT_1_C Calc. and Reported MoietyFormula Strings  Differ     Please Check
              Calc: C25 H10 Ag3 F18 N7 O2
              Rep.: C15 H3 Ag3 F18 N6, C10 H7 N O2
PLAT213_ALERT_2_C Atom F00Q            has ADP max/min Ratio .....         3.4 prolat
PLAT213_ALERT_2_C Atom F01J            has ADP max/min Ratio .....         3.4 prolat
PLAT213_ALERT_2_C Atom F1              has ADP max/min Ratio .....         3.2 prolat
PLAT213_ALERT_2_C Atom F01K            has ADP max/min Ratio .....         3.4 prolat
PLAT213_ALERT_2_C Atom F01C            has ADP max/min Ratio .....         3.9 prolat
PLAT220_ALERT_2_C NonSolvent   Resd 1  F   Ueq(max)/Ueq(min) Range         3.6 Ratio
PLAT906_ALERT_3_C Large K Value in the Analysis of Variance ......       2.035 Check
PLAT911_ALERT_3_C Missing FCF Refl Between Thmin & STh/L=    0.600          37 Report
                 2  0  0,  -3  1  0,  -2  1  0,  -1  1  0,  -5 -2  1,   0 -1  1,
                -1  0  1,   1  0  1,   8 -9  2,  10 -3  2,  10 -2  2,  -5 -1  2,
                10 -1  2, -10  3  2, -10  4  2,   9 -2  3,   9 -1  3,   0  2  3,
               -10  3  3,   9 -5  4,   9 -3  4,   9 -2  4,   9 -1  4,  -1  1  4,
                -1  2  4,  -5 -6  5,   9 -2  5,   9 -1  5,   9 -2  6,   9 -3  7,
                    (    7 More Missing: see the .ckf listing file)
```

Alert level G

```
PLAT002_ALERT_2_G Number of Distance or Angle Restraints on AtSite           7 Note
PLAT004_ALERT_5_G Polymeric Structure Found with Maximum Dimension           1 Info
PLAT083_ALERT_2_G SHELXL Second Parameter in WGHT  Unusually Large        7.17 Why ?
PLAT154_ALERT_1_G The s.u.'s on the Cell Angles are Equal ..(Note)       0.002 Degree
PLAT176_ALERT_4_G The CIF-Embedded .res File Contains SADI Records           3 Report
PLAT242_ALERT_2_G Low    'MainMol' Ueq as Compared to Neighbors of        C01A Check
PLAT242_ALERT_2_G Low    'MainMol' Ueq as Compared to Neighbors of        C01C Check
PLAT242_ALERT_2_G Low    'MainMol' Ueq as Compared to Neighbors of        C01D Check
PLAT242_ALERT_2_G Low    'MainMol' Ueq as Compared to Neighbors of        C01G Check
PLAT242_ALERT_2_G Low    'MainMol' Ueq as Compared to Neighbors of        C01H Check
PLAT301_ALERT_3_G Main Residue  Disorder .............(Resd    1)           5% Note
PLAT434_ALERT_2_G Short Inter HL..HL Contact F005      ..F00P      .      2.82 Ang.
                                                  x,1+y,z  =      1_565 Check
PLAT720_ALERT_4_G Number of Unusual/Non-Standard Labels .........           67 Note
              Ag01    Ag02    Ag03    F004    F005    F006    F007    F008
              N009    F00A    O00B    N00C    O00D    N00E    F00F    F00G
              N00H    F00I    F00J    N00K    F00L    N00M    F00N    F00O
              F00P    F00Q    N00R    C00S    H00S    C00T    C00U    C00V
              C00W    H00W    C00X    C00Y    H00Y    C00Z    C010    C011
              C012    H012    C013    C014    H014    C015    C016    C017
              H017    C018    H018    C019    H019    C01A    F01B    C01C
              C01D    C01E    H01E    C01F    H01F    C01G    C01H    F01I
```

```
              F01J    F01K    F01C
PLAT860_ALERT_3_G Number of Least-Squares Restraints ............          3 Note
PLAT883_ALERT_1_G Absent Datum for _atom_sites_solution_primary ..     Please Do !
PLAT912_ALERT_4_G Missing # of FCF Reflections Above STh/L=  0.600       314 Note
PLAT913_ALERT_3_G Missing # of Very Strong Reflections in FCF ....         3 Note
                2  0  0,  -3  1  0,  -1  2  4,
PLAT933_ALERT_2_G Number of HKL-OMIT Records in Embedded .res File         4 Note
              -10  3  2, -10  4  2,   8 -9  2, -10  3  3,
PLAT941_ALERT_3_G Average HKL Measurement Multiplicity ..........        2.1 Low
PLAT969_ALERT_5_G The 'Henn et al.' R-Factor-gap value ..........      3.344 Note
              Predicted wR2: Based on SigI**2  3.30 or SHELX Weight 10.47
PLAT978_ALERT_2_G Number C-C Bonds with Positive Residual Density.         0 Info
```

```
   0 ALERT level A = Most likely a serious problem - resolve or explain
   0 ALERT level B = A potentially serious problem, consider carefully
   9 ALERT level C = Check. Ensure it is not caused by an omission or oversight
  21 ALERT level G = General information/check it is not something unexpected

   3 ALERT type 1 CIF construction/syntax error, inconsistent or missing data
  16 ALERT type 2 Indicator that the structure model may be wrong or deficient
   6 ALERT type 3 Indicator that the structure quality may be low
   3 ALERT type 4 Improvement, methodology, query or suggestion
   2 ALERT type 5 Informative message, check
```

Validation response form

Please find below a validation response form (VRF) that can be filled in and pasted into your CIF.

```
# start Validation Reply Form
_vrf_PLAT042_lcz080
;
PROBLEM: Calc. and Reported MoietyFormula Strings  Differ     Please Check
RESPONSE: ...
;
_vrf_PLAT213_lcz080
;
PROBLEM: Atom F00Q            has ADP max/min Ratio .....        3.4 prolat
RESPONSE: ...
;
_vrf_PLAT220_lcz080
;
PROBLEM: NonSolvent   Resd 1  F   Ueq(max)/Ueq(min) Range        3.6 Ratio
RESPONSE: ...
;
_vrf_PLAT906_lcz080
;
PROBLEM: Large K Value in the Analysis of Variance ......      2.035 Check
RESPONSE: ...
;
_vrf_PLAT911_lcz080
;
PROBLEM: Missing FCF Refl Between Thmin & STh/L=    0.600         37 Report
RESPONSE: ...
;
# end Validation Reply Form
```

It is advisable to attempt to resolve as many as possible of the alerts in all categories. Often the minor alerts point to easily fixed oversights, errors and omissions in your CIF or refinement strategy, so attention to these fine details can be worthwhile. It is up to the individual to critically assess their own results and, if necessary, seek expert advice.

PLATON version of 17/09/2025; check.def file version of 17/09/2025

---

duplicate check

No duplication found

Datablock lcz080 - ellipsoid plot

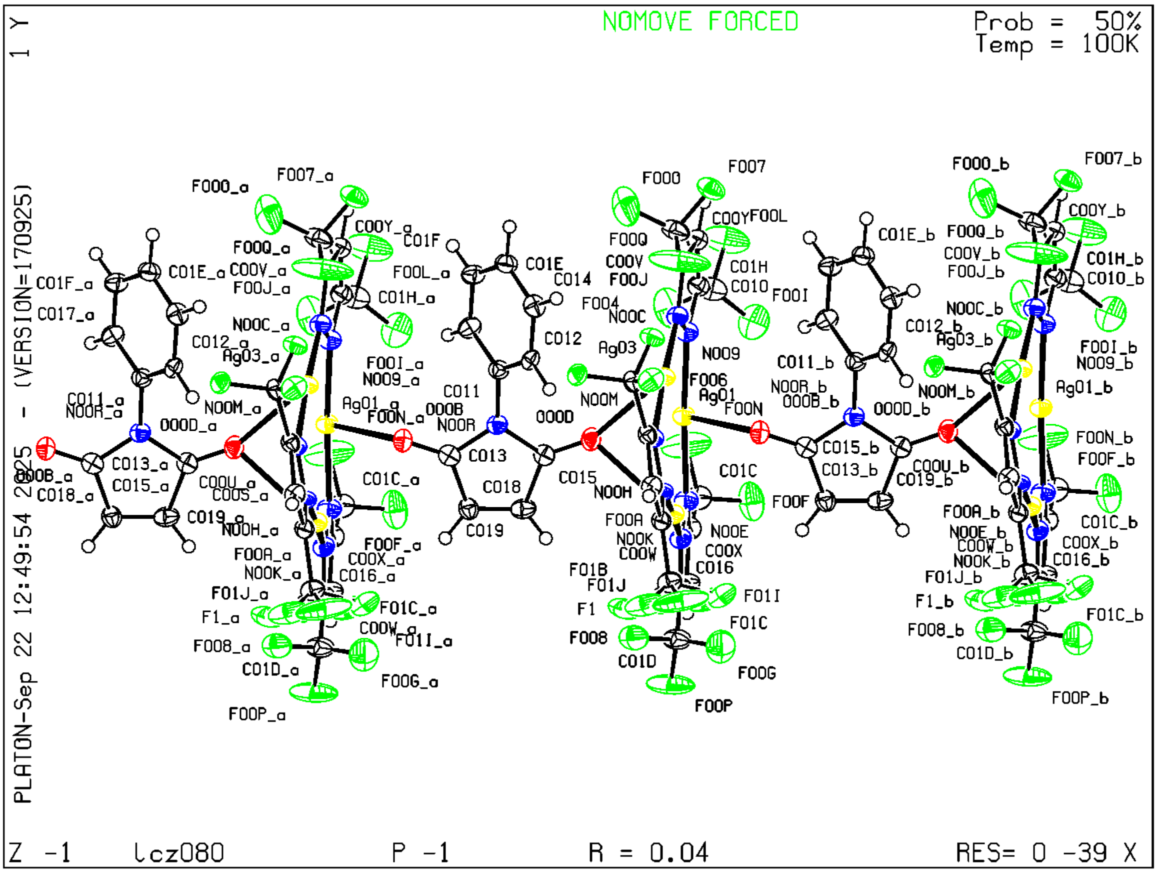

Supplement: Supplementary file 2 — Supporting File 2: anie72462‐sup‐0002‐DataFile.zip. [file ANIE-65-e1218503-s002.zip › supplementary_materials_crystallographic_data submit.zip!/cif_and_checkcif(1-114)/Ag3Pz3_80/Ag3Pz3_80_checkcif.pdf]
